# Tuberculosis control in the Republic of Korea

**DOI:** 10.4178/epih.e2018036

**Published:** 2018-08-02

**Authors:** Kyung Sook Cho

**Affiliations:** Division of Social Services Projects, Office of Social Welfare Policy, Ministry of Health and Welfare, Sejong, Korea

**Keywords:** Tuberculosis, Latent tuberculosis, Incidence, Mortality, Interferon-gamma release tests, Republic of Korea

## Abstract

The incidence and mortality rates of tuberculosis (TB) in the Republic of Korea are 77 and 5.2 per 100,000 people, respectively (2016), which are the highest among the member countries of the Organization for Economic Cooperation and Development. Recently, the incidence of TB among teens and individuals in their 20s in the Republic of Korea decreased significantly. The decrease is largely attributed to the TB screening and contact investigation efforts targeting schools over the past few years. However, the incidence of TB among elderly individuals remains high, and it is even increasing compared to that in the past 10 years. Older individuals account for 42% of all TB cases and 82% of TB-related deaths. The success rate of TB treatment in the Republic of Korea has gradually increased due to various programs, such as control of non-compliance, insurance coverage for TB diagnosis and treatment, and TB public–private mix models. This study suggests that policy makers should focus their efforts on policies that prioritize a significant reduction in the incidence of TB based on the 2nd National Strategic Plan for Tuberculosis Control (2018–2022).

## INTRODUCTION

Tuberculosis (TB) has an extremely high global disease burden with more than 10 million new TB cases and approximately 1.7 million TB-related deaths reported annually worldwide [1]. In the Republic of Korea (hereafter Korea), 28,161 new TB cases were reported and the total number of notified patients with TB was 36,044 in 2017. In addition, approximately 2,200 TB-related deaths were reported in 2016 [2].

Since the initial implementation of the national TB control program in health centers throughout Korea in 1962, the government of Korea has legislated the TB prevention law in 1968 to establish the legal basis for the national TB control program. In 1989, a transition from health center- to private hospital-based TB control program began with the introduction of the nationwide health insurance system, and since 2010, significant effort has focused on TB public–private mix (PPM) model. Recently, TB contact investigation has been reinforced and conducted on family members and other individuals who are in close contact with patients with TB as an effort for the early detection of such condition, and this measure is in addition to the significant effort made in the treatment and management of patients with TB, including free TB diagnosis and treatment and allocation of TB-specialist nurses [3]. Consequently, the prevalence, incidence, and mortality rate associated with TB have steadily decreased over the past few decades. Despite such effort, Korea still had high incidence and mortality rates of TB (77 and 5.2 per 100,000 people, respectively) as of 2016, which represents the highest incidence and mortality rates of TB among all member countries of the Organization for Economic Cooperation and Development (OECD) [1].

Accordingly, the government of Korea is planning to establish the 2nd National Strategic Plan for Tuberculosis Control (2018–2022) to implement more intensive TB control policies. Thus, the present study aimed to analyze the incidence, prevalence, and mortality rates of TB in Korea as well as the various TB control policies implemented by the government of Korea over the past few decades to present future improvement measures.

## TUBERCULOSIS IN KOREA

### Prevalence, incidence, and mortality rates of tuberculosis

**Figure 1.**
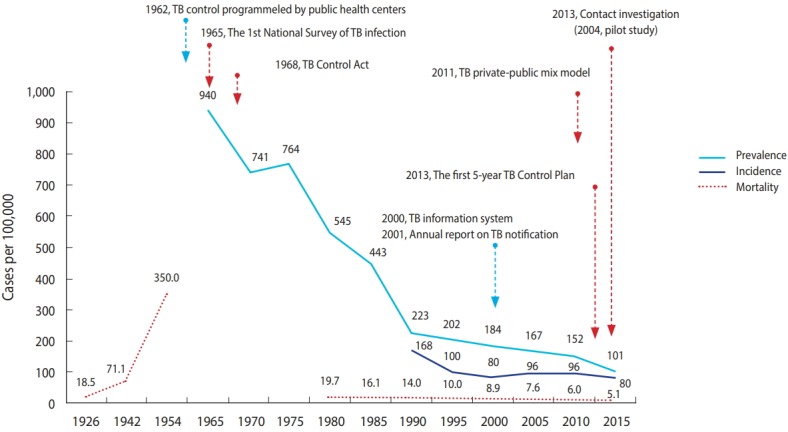
Annual prevalence, incidence, and mortality rate of tuberculosis (TB). Adapted from Cho KS. Health Soc Welf Rev 2017;37:179-212 [3], with permission of the Korea Institute for Health and Social Affairs.

The mortality rates of TB in Korea were approximately 18.5 and 71.1 per 100,000 population in 1926 and 1942, respectively. However, it increased sharply to 350.0 per 100,000 in 1954 after the Korean War. The mortality rate decreased from 19.7 per 100,000 population in 1983 to 8.9 per 100,000 population in 2000, and as of 2015, the rate is 5.1 per 100,000 population. The incidence rate of TB has decreased by approximately half over a 25-year period (from 168 per 100,000 population in 1990 to 80 per 100,000 population in 2015). The prevalence of TB has also decreased by approximately half (from 940 per 100,000 population in 1965 to 443 per 100,000 population in 1985), and for the following 30 years, the rate decreased to one-quarter of the level to just 101 per 100,000 population in 2015. In particular, the sharp decrease in the prevalence of TB up to 1990 became somewhat sluggish until 2010, and after which, the rate increased sharply again (Figure 1 and Supplementary Material 1).

### Notified tuberculosis cases and deaths

In Korea, TB has been considered a legal communicable disease since 1957 in accordance with the Infectious Disease Control and Prevention Act, which made its reporting mandatory. In 1968, reporting and registration became mandatory based on the Tuberculosis Prevention Act, whereas the requirement was made more stringent in 2003 with mandatory notification. In 2000, the Tuberculosis Notification Information System was established, requiring all patients with TB to electronically notify and register cases starting in 2001. The total TB cases notified in 2017 were 36,044, representing approximately 22% decrease from 46,082 cases in 2001. The number of new notified TB cases in 2017 was 28,161, representing approximately 21% decrease from 34,123 cases in 2001 [2]. In addition, the rate of total notified TB cases decreased from 96.3 per 100,000 population in 2001 to 70.4 per 100,000 population in 2017. The rate of new notified TB cases also decreased from 71.3 per 100,000 population in 2001 to 55.0 per 100,000 population in 2017, and this showed a decreasing trend in all age groups, except the ≥80-year age group. Compared to other age groups, the 10-19- and 20-29-year age groups showed a more steep decreasing trend, and in particular, the ≤19-year age group showed a decrease to just one-third of the level in 2001 [2,4] (Supplementary Materials 2 and 3). With respect to the total notified TB cases in 2017 based on gender, the percentage of men (60.1%) was significantly higher than that of women (39.9%), whereas the percentage of new notified TB cases was also higher in men (57.3%) than in women (42.7%) [2]. Among the total notified TB cases, the percentage accounted for by elderly individuals aged ≥65 years increased from 20.2% in 2001 to 41.9% in 2017. The number of TB-related mortalities decreased from 3,218 in 2001 to 2,186 in 2016. However, among the total TB-related deaths, the proportion of elderly individuals aged ≥65 years increased from 58.0% in 2001 to 81.7% in 2016 [2] (Supplementary Materials 4).

## TUBERCULOSIS CONTROL IN KOREA

### Changes in the tuberculosis control programs

**Figure 2.**
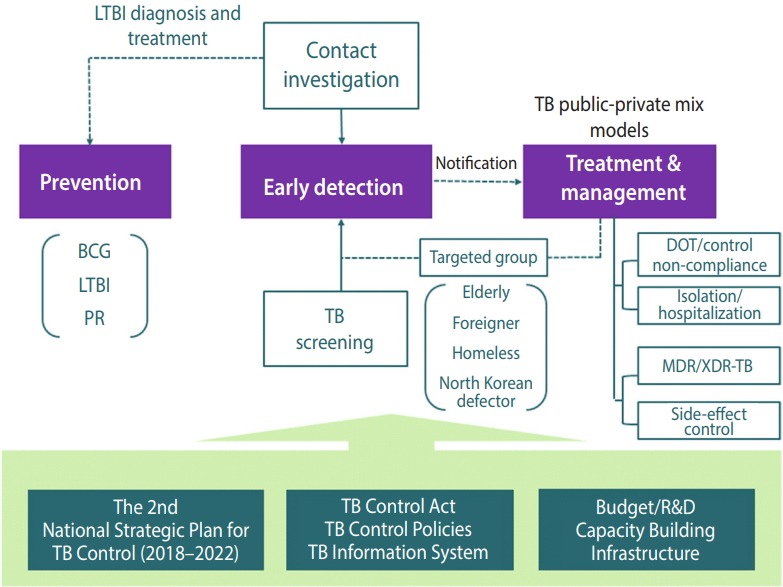
TB control framework of the Republic of Korea. TB, tuberculosis; LTBI, latent TB infection; BCG, bacille de Calmette-Guerin vaccine; PR, public relation; DOT, directly observed treatment; MDR, multidrug-resistant; XDR, extensively drug-registant. Adapted from Cho KS. Health Soc Welf Rev 2017;37:179-212 [3], with permission of the Korea Institute for Health and Social Affairs.

Korea began its TB control programs in 1962, focusing on early detection and treatment in health centers located nationwide. Such TB control policy efforts included the following. Starting from 1965, National TB Prevalence Surveys have been conducted every 5 years, and since 2001, Annual reports on the notified TB have been published by notifying and registering all patients with TB in accordance with the Tuberculosis Prevention Act. In 1968, the Tuberculosis Prevention Act was enacted to mark the beginning of a full-fledged implementation of government-led TB control policies. In 2006, the Tuberculosis Elimination Plan by 2030 was established, and in 2013, the National Strategic Plan for Tuberculosis Control was established every 5 years. In particular, the TB PPM model was developed in 2011, and the focus was on the care of patients with TB. That is, TB-specialist nurses were dispatched to 252 health centers and approximately 120 health care facilities throughout Korea. In 2004, TB contact investigation began. By 2009, the budget for TB contact investigation was allocated, and a central epidemiological investigation team has been conducting an official TB contact investigation since 2013. In addition, free government-sponsored TB diagnosis and treatment services are being provided since 2011. In 2017, latent TB infection (LTBI) screening and treatment services were provided to approximately 1.2 million individuals. In 2018, TB screening pilot program for elderly individuals and TB and latent TB screening pilot programs for foreigners were implemented with plans to intensify programs for the vulnerable population that had been neglected until currently (Figure 1). The TB control framework in Korea is shown in Figure 2. Tuberculosis Prevention Act, National Strategic Plan for Tuberculosis Control, and research and development efforts are established based on national TB control policies. Also, various policies and programs are being operated in terms of prevention, early detection, and patient treatment and management (Figure 2).

### Tuberculosis budget

The budget for national TB control program, which is a national expenditure, increased sharply from 9.9 billion Korean won (KRW) in 2007 to 14.9 billion KRW in 2010 and to 44.7 billion KRW in 2011, and this reflects the fact that government subsidy for personal co-payment amounting to 10% of the health insurance TB treatment cost began in 2011. Such co-payment for TB treatment was waived in 2017, and it is paid in full by health insurance. In 2017, 8.9 billion KRW of relevant budget was paid for the LTBI screening program for healthcare workers, kindergarten teachers, nursery workers, workers in social welfare facilities in accordance with the Tuberculosis Prevention Act, and this accounts for the large margin of increase in expenditure for TB and LTBI early detection programs. However, LTBI screening and treatment was only a 1-year program conducted in 2017 based on the amendment of the law. In 2018, LTBI screening and treatment fees for medical personnel were partially covered, and despite the budgets allocated for new programs in 2018 (620 million KRW for TB screening programs for elderly individuals and 450 million KRW for foreign TB/LTBI screening programs), the total budget was only 34.3 billion KRW (Table 1).

### Tuberculosis prevention

Despite the high incidence rate of TB in Korea, public interest in and understanding of TB are extremely low. Accordingly, the government of Korea implemented various promotional activities for improving knowledge about TB, encouraging early TB screening, and proper coughing etiquette. In Korea, TB vaccination is mandatory and budget support is provided by the government. The vaccination rate for TB has increased from 16.4% in 1965 to 65.7% in 1990 and from 87.0% in 2003 to 99.8% in 2013 [6,7]. Korea relies solely on imported TB vaccines (bacille de Calmette-Guerin vaccine [BCG] vaccine). However, to resolve the unstable supply of TB vaccines that occurs at certain times and to provide a timely supply of such vaccines, in-country development efforts began in 2014 with 2020 as the target completion date for TB vaccine development (BCG vaccine).

The prevalence of LTBI is extremely high at 33% in Korea [3,8] (Supplementary Materials 1 and 5). In response to this, prevention is being promoted through LTBI diagnosis and treatment for high-risk groups. For individuals infected with human immunodeficiency virus, TB contacts, organ transplant recipients, tumor necrosis factor users, patients on dialysis, and those with silicosis, who are strongly recommended for systematic LTBI diagnosis and treatment by the World Health Organization (WHO) [9], LTBI screening is provided as medical care benefit under the health insurance [3,10]. Moreover, TB and LTBI screening became mandatory for healthcare workers, postnatal care workers, nursery workers, workers in children welfare facilities, and teachers at kindergartens and primary, secondary, and high schools since 2017 in accordance with the Tuberculosis Prevention Act; the government of Korea paid for systematic LTBI screening and treatment for approximately 1.2 million individuals within this target population [3,10].

### Early detection of tuberculosis

In Korea, TB screening (chest radiography) is performed as a part of the health examination conducted on children who are entering secondary or high school. For adults, TB screening is included in the health examination performed every 1-2 years for health insurance subscribers. Moreover, the Tuberculosis Prevention Act makes it mandatory for healthcare workers, teachers, nursery workers, and workers in postnatal care centers and children welfare facilities to receive regular TB and LTBI screening. However, the vulnerable population, including elderly individuals, foreigners, and the homeless, have been in the blind spot of TB screening. In response to this, the government of Korea is planning to implement TB screening pilot programs for elderly individuals and TB and LTBI screening pilot programs for foreigners starting in 2018 to establish a systematic early detection strategy for this population.

Another important aspect of early TB detection is TB contact investigation, and in accordance with the Tuberculosis Prevention Act, TB contact investigation is being conducted on household contacts of TB cases and individuals who are in close contact with patients with TB in schools, hospitals, workplaces, social welfare facilities, armies, and prisons. Beginning with 13 cases in the TB contact investigation in 2004, the number of cases increased to 1,142 in 2013 after the program was fully implemented. Subsequently, the number of cases continued to increase with 1,405, 2,639, and 3,502 cases in 2014, 2015, and 2016, respectively (Supplementary Material 6). In 2016, the number of individuals targeted for TB contact investigation was 146,911, of whom 12,707 were diagnosed with LTBI [11] (Supplementary Material 7).

### Treatment and management of tuberculosis

In Korea, the notification and registration of all patients with TB are mandatory according to the Tuberculosis Prevention Act. The government is providing diagnosis and treatment services at no cost as well as care until a patient is completely cured. Moreover, the national TB control program also provides financial support for LTBI screening and treatment. Among individuals who were diagnosed with TB in Korea, workers or students are restricted from going to work or school until they are no longer contagious, and they are followed-up to make sure they were under medication for at least 2 weeks. Since 2013, directly observed treatment is being recommended in Korea. However, it still fails to meet the guidelines by the WHO. In addition, treatments for patients who are non-compliant to treatment are being managed through telephone consultation and home visits. The treatment success rates for such non-compliant patients with TB have improved from 56.4% in 2014 to 50.0% in 2015 and to 63.1% in 2016. The total treatment success rate for patients with TB in 2016 was 84.3% for all patients with TB, 86.7% for new patients with TB, and 83.3% for patients with new smear-positive TB. These indicators are showing gradual improvement annually [3].

Approximately 3.4% of new patients were diagnosed with rifampicin-resistant (RR) and multidrug-resistant (MDR) TB (RR/MDR-TB) in Korea, which is higher than the average of 2.6% among the member countries of the OECD. The repeat patient rate is 11%, which is slightly lower than the average of 12% among the member countries of the OECD [12]. In particular, the percentage of foreigners with MDR-TB in Korea increased from 4.5% in 2011 to 20.5% in 2016 (Supplementary Material 8 and 9). To increase the treatment success rate of patients with MDR-TB in Korea, new high-priced MDR-TB drugs have recently been added to health insurance coverage, and these drugs are being provided at no cost. The treatment success rate of patients with RR/MDR-TB in Korea is 63%, which is similar to the average of 62% among the member countries of the OECD [12].

One of the most successful models for the treatment and management of patients with TB is the TB PPM model in Korea. After the pilot program in 2007, this model was expanded nationwide in 2011, contributing to the improvements in the treatment success rate for patients with TB through organization cooperation between 252 health centers and approximately 120 general hospitals, wherein 420 TB management specialist nurses have been dispatched to such health centers and hospitals to conduct systematic TB control program, including TB patient consultation, medication guidance, and non-compliant patient management.

## DISCUSSION

The incidence and mortality rates of TB in Korea reached their peak after the Korean War in 1950 and subsequently showed a rapid decrease (Figure 1). However, the rates are still the highest among all member countries of the OECD [1,2]. The high incidence of TB in Korea despite its high economic standards may be attributed to the Korean War. The outbreak of TB after a war has been observed in other countries as well [13,14], and a significant portion of the Korean population with poor health conditions, such as malnutrition, dense living environment, and low health care accessibility, may have been infected by LTB after the war [3,5]. The LTBI rate in Korea has decreased steadily from 64.2% in 1960 to 59.3% in 1975, to 44.4% in 1990, and to 33.2% in 2016 (Supplementary Material 5). However, the rate is still higher than that of other advanced countries, such as the US [3,8,15]. The high LTBI rate can be considered as one of the biggest barriers in reducing TB in Korea. In 2017, the government of Korea amended the Tuberculosis Prevention Act to make TB and LTBI screening mandatory for specific groups such as health care and postpartum care workers and teachers. Moreover, systematic LTBI screening and treatment were conducted for 1.2 million individuals [3,10] (Supplementary Material 5 and Supplementary Material 10).

Recently, the treatment success rate for patients with TB in Korea has improved due to timely diagnosis and treatment. Of which, patient treatment and management based on the TB PPM model have made a significant contribution, leading to a continuous decrease in the incidence of TB [3]. Moreover, a more drastic decrease was observed in the number of patients with TB in the ≤19-year age group than in any other age groups, which may be attributed to the fact that individuals in this age group were born during the period when the incidence of TB was gradually decreasing and full-scale TB contact investigation and TB screening are being conducted at schools [3,4]. However, the incidence of TB among elderly individuals (≥65 years of age) remains high, and the elderly population accounts for 42% of all patients with TB and 82% of all TB-related deaths in Korea. The elderly population shows a lower TB screening rate than the other age groups, and TB control programs for elderly individuals are limited as well. To address such problems, the government of Korea is planning to establish TB control programs for elderly individuals by implementing pilot programs for TB screening and early detection in 2018. By contrast, TB screening is mandatory for foreigners who are from 19 countries with a high incidence rate of TB and enter Korea for residency or work purposes. However, the management of TB among these foreigners after they have entered Korea is difficult. Accordingly, the government of Korea is also planning to launch a pilot program that examines the performance of TB and LTBI screening among foreigners during their stay in Korea.

The government of Korea established the 2nd National Strategic Plan for Tuberculosis Control (2018–2022) with the goal of lowering the incidence rate of TB from 77 to 40 per 100,000 population by 2022. For the next 5 years, the government of Korea should faithfully execute this plan and allocate the necessary budget according to priorities. First, the top priority for TB control in Korea should be the early diagnosis and treatment of patients with TB. For timely diagnosis and improved treatment rate, health insurance coverage related to TB diagnosis and treatment should be expanded. In addition, to allow timely diagnosis and standardized treatment to be provided at primary care facilities, the reinforcement of the TB PPM program that focuses on medical personnel education and intensive care for patients with TB is also necessary (Supplementary Material 11). Furthermore, research and development efforts for shortening the treatment period and improving the treatment success rate for patients with MDR TB must be expanded. By contrast, measures for the early detection and systematic treatment of TB among the vulnerable population, including elderly individuals, foreigners, and the homeless, who have been neglected until currently, should be explored. With respect to TB treatment for the vulnerable population, a community-based comprehensive public health patient care model should be developed and implemented, whereas greater resources, such as isolation unit for patients with TB and allocation of TB specialist nurses, are also in demand. Second, TB contact investigation should be reinforced to establish an optimal investigation system tailored for collective facilities, such as schools, hospitals, and prisons. The contact investigation should prioritize those in close contact over those in casual contact with individuals with TB, whereas effort is also needed to increase the screening rate for those being tested and treatment success rate for those who tested positive for LTBI [4,12,16-20]. Third, from the TB prevention aspect, promotional strategies must be established for regular TB screening and the timely supply of vaccines by successfully completing the development of TB vaccine that has undergone repeated supply shortage. Moreover, LTBI screening and treatment for high-risk groups including healthcare workers must be continually conducted. Longterm follow-up is needed for LTBI screening and treatment that were performed on 1.2 million individuals in 2017 [12].

## Figures and Tables

**Table 1.** TB budgets of central government by year

Category	2007	2008	2009	2010	2011	2012	2013	2014	2015	2016	2017	2018
TB prevention and Infrastructure	4.4	4.1	1.6	1.9	1.9	2.3	2.7	2.3	2.5	2.6	2.6	2.7
TB treatment and management	0.2	0.2	5.5	6.6	33.6	18.6	18.5	19.6	20.5	19.7	12.7	13.1
TB and LTBI screening	NA	NA	0.6	0.6	8.0	10.3	12.2	11.0	10.6	12.9	20.8	14.1
R&D and others	5.3	7.7	4.7	5.8	1.2	7.9	5.7	3.6	3.4	4.1	5.1	4.4
Total	9.9	12.0	12.4	14.9	44.7	39.1	39.1	36.5	37.0	39.3	41.2	34.3

Unit: 10^9^ Korean won (1,000 Korean won = 1 US dollar). TB, tuberculosis; LTBI, latent TB infection; NA, not available; R&D, research and development. Adapted from Cho KS. Health Soc Welf Rev 2017;37:179-212 [3], with permission of the Korea Institute for Health and Social Affairs.

## SUPPLEMENTARY MATERIALS

Supplementary Material 1: Table S1 is available at http://www.e-epih.org/).

Supplementary Material 1: Table S1 is available at http://www.e-epih.org/).

Supplementary Material 2: Figure S1 is available at http://www.e-epih.org/).

Supplementary Material 3: Figure S2 is available at http://www.e-epih.org/).

Supplementary Material 4: Figure S3 is available at http://www.e-epih.org/).

Supplementary Material 5: Table S2 is available at http://www.e-epih.org/).

Supplementary Material 6: Table S3 is available at http://www.e-epih.org/).

Supplementary Material 7: Figure S4 is available at http://www.e-epih.org/).

Supplementary Material 8: Table S4 is available at http://www.e-epih.org/).

Supplementary Material 9: Figure S5 is available at http://www.e-epih.org/).

Supplementary Material 10: Table S5 is available at http://www.e-epih.org/).

Supplementary Material 11: Figure S6 is available at http://www.e-epih.org/).
