# Supplementary material for: Tuberculosis control in the Republic of Korea
Source: Epidemiol Health. 2018 Aug 2;40:e2018036. doi: 10.4178/epih.e2018036 (PMC6335497; doi:10.4178/epih.e2018036)
Supplement: Supplementary file 1 [file epih-40-e2018036-supplementary.pdf]

Volume: 40, Article ID: e2018036
https://doi.org/10.4178/epih.e2018036

한국의 결핵관리 현황

조경숙

보건복지부 사회서비스정책관 사회서비스사업과

Tuberculosis Control in the Republic of Korea

Kyung Sook Cho

Division of Social Services Projects, Office of Social Welfare Policy, Ministry of Health and Welfare, Sejong, Republic of Korea

Corresponding Author: Kyung Sook Cho
Division of Social Services Projects, Office of Social Welfare Policy, Ministry of Health and Welfare, Doum4-ro, Sejong Special Self-Governing City 30113, Republic of Korea
E-mail: ks.g.cho@gmail.com

이 원고는 한국보건사회연구원의 승인을 받아 '우리나라 결핵실태 및 국가 결핵관리 현황'이라는 제목으로 보건사회연구(2017;37(4):179-212)에 게재된 논문을 수정 및 보완한 것입니다.

Volume: 40, Article ID: e2018036
https://doi.org/10.4178/epih.e2018036

Abstract

The incidence and mortality rates of tuberculosis (TB) in the Republic of Korea are 77 and 5.2 per 100,000 people, respectively (2016), which are the highest among the member countries of the Organization for Economic Co-operation and Development. Recently, the incidence of TB among teens and individuals in their 20s in the Republic of Korea decreased significantly. The decrease is largely attributed to the TB screening and contact investigation efforts targeting schools over the past few years. However, the incidence of TB among elderly individuals remains high, and it is even increasing compared to that in the past 10 years. Older individuals account for 42% of all TB cases and 82% of TB-related deaths. The success rate of TB treatment in the Republic of Korea has gradually increased due to various programs, such as control of noncompliance, insurance coverage for TB diagnosis and treatment, and TB public–private mix models. This study suggests that policy makers should focus their efforts on policies that prioritize a significant reduction in the incidence of TB based on the 2nd National Strategic Plan for Tuberculosis Control (2018–2022).

Keywords: Tuberculosis, Latent Tuberculosis, Incidence, Mortality, Interferon-gamma Release Tests, Republic of Korea

서론 (INTRODUCTION)

전 세계적으로 매년 1 천만 명 이상의 결핵 환자가 신규로 발생하고, 연간 170 만여 명이 결핵으로 사망하고 있는 등 결핵은 전 세계적으로 질병부담이 매우 높은 질병 중 하나이다[1]. 한국에서는 연간 2 만 8 천여 명의 결핵 신 환자가 신고되고 있고, 신고된 총 결핵환자수는 3 만 6 천여 명에 이르며, 연간 2 천 2 백여 명이 결핵으로 사망하고 있다[2].

한국은 지난 1962 년에 전국 보건소에서 결핵관리 사업을 시작한 이래, 1968 년에 결핵예방법을 제정하여 결핵관리의 법적 근거를 마련하였다. 1989 년에는 전 국민 의료보험이 도입됨에 따라 보건소 중심의 결핵관리에서 민간 병원 중심의 결핵관리로 변화되기 시작하였고, 2010 년 이후에는 민간과 공공이 협력하는 결핵관리사업(PPM, TB public-private mix)에 심혈을 기울여 오고 있다. 최근에는 결핵환자와 접촉한 가족 및 밀접 접촉자에 대한 결핵 접촉자 조사(TB contact investigation)를 강화하여 결핵 조기발견에 힘쓰고 있을 뿐 아니라, 결핵 진단과 치료 비용을 국가에서 무료로 제공하고 결핵 전담 간호사를 배치하는 등 결핵환자 치료 및 관리에 많은 노력을 기울이고 있다[3]. 이로 인해 지난 수십 년 동안 결핵 유병률과 발생률, 그리고 사망률은 꾸준히 감소해 왔다. 그럼에도 불구하고 한국은 2016년 기준으로 인구 10만 명당 77 명이라는 높은 결핵 발생률과 인구 10 만 명당 5.2 명이라는 높은 결핵 사망률을 보여, 여전히 경제협력개발기구(OECD, Organization for Economic Cooperation and Development) 회원국 중 결핵 발생과 사망에서 가장 높은 수준을 보이고 있다[1].

이에 한국 정부는 제 2 기 결핵관리 종합계획(2018~2022)을 수립하여 보다 강도 높은 결핵관리정책을 시행하려 하고 있다. 본 연구에서는 한국의 결핵 발생률, 유병률, 사망률에 대한 분석과 더불어, 지난 수 십 년 동안 한국 정부가 시행해 온 다양한 결핵관리 정책들을 분석하고 향후 개선방안을 제시하고자 하였다.

Volume: 40, Article ID: e2018036
https://doi.org/10.4178/epih.e2018036

한국의 결핵 현황 (TUBERCULOSIS IN THE REPUBLIC OF KOREA)

결핵 유병률, 발생률 및 사망률 (TB Prevalence, Incidence, and Mortality)

한국의 1926 년 결핵 사망률은 인구 10 만 명당 18.5 명으로 추정되며, 1942 년 71.1 명, 한국전쟁 이후인 1954 년에는 350 명으로 급격히 증가한 것으로 추정해 볼 수 있다. 이러한 결핵 사망률은 1983 년 19.7 명에서 2000 년 8.9 명으로 감소하였고, 2015 년에는 5.1 명 수준이었다. 결핵 발생률의 경우는 1990 년 인구 10 만 명당 168 명에서 2015 년 80 명 수준으로 25 년간 절반 수준으로 감소하였고, 결핵 유병률은 1965 년 인구 10 만 명당 940 명 수준에서 1985 년 443 명으로 20 년간 절반 수준으로 감소하였으며, 2015 년에는 101 명으로 이후 30 년 동안 4 분의 1 수준으로 급격히 감소하였다. 특히, 1990 년까지 급격히 감소하던 결핵 유병률은 2010 년까지 그 감소율이 다소 둔화되었다가 2010 년 이후 다시 감소 속도가 빨라지고 있다(그림 1).

신고 결핵 환자 및 결핵 사망 현황 (Notified TB Cases and TB Deaths)

한국에서 결핵은 1957 년부터 전염병예방법에 따라 법정전염병으로 지정되어 보고가 의무화되었고, 1968 년부터는 결핵예방법에 따라 보고 및 등록이 의무화되었으며, 2003 년부터는 신고의무로 강화되었다. 2000 년에는 결핵 신고 정보시스템을 구축하고 2001 년부터 전산으로 결핵환자를 전수 신고·등록하도록 하고 있다. 2017 년 신고 결핵 전체 환자수(total TB cases notified)는 36,044 명으로 2001 년 46,082 명에 비해 약 22% 감소하였고, 2017 년 신고 결핵 신환자수(new TB notification cases)는 28,161 명으로 2001 년 34,123 명 비해 약 21% 감소하였다[2]. 신고 결핵 전체 환자율로 살펴보면, 2001 년 인구 10 만 명당 96.3 명에서 2017 년 70.4 명으로 감소하였고, 신고 결핵 신환자율은 2001 년 인구 10 만 명당 71.3 명에서 2017 년 55.0 명으로 감소하였으며, 이는 80 세 이상 연령층에서의 증가 추세를 제외하고는 전 연령층에서 감소 추세를 보였다. 다른 연령층에 비해 10 대 및 20 대에서 급격한 감소세를 보였고, 특히 19 세

Volume: 40, Article ID: e2018036
https://doi.org/10.4178/epih.e2018036
이하의 연령층에서는 지난 2001 년에 비해 1/3 수준으로 감소하였다[2,4]. 2017 년 성별에 따른 신고결핵 전체 환자 비율은 살펴보면, 남자 60.1%, 여자 39.9%로 남자의 비중이 월등히 높았으며, 신고결핵 신환자중 남자의 비율은 57.3%, 여자는 42.7%로 이 역시 남자의 비중이 높았다[2]. 한편, 신고 결핵 전체 환자에서 65 세 이상 노인이 차지하는 비중은 2001 년 20.2%에서 2017 년 41.9%로 증가하였다. 결핵 사망자수는 2001 년 3,218 명에서 2016 년 2,186 명으로 감소 추세에 있으나, 전체 결핵 사망자중 65 세 이상 노인의 비중은 2001 년 58.0%에서 2016 년 81.7%로 증가하였다[2].

한국의 결핵 관리 정책 현황 (TB CONTROL IN THE REPUBLIC OF KOREA)

결핵 관리 사업의 변천 과정 (Changes of TB Control Programs)

한국은 1962 년 전국 보건소를 통해 환자 조기 발견 및 치료 중심의 결핵관리사업을 시작하였다. 1965 년부터는 5 년마다 결핵 실태 조사를 실시하였고, 2001 년부터는 결핵예방법에 따라 결핵 환자를 전수 신고·등록하도록 하여 매년 결핵 신고 현황 보고서를 발간하고 있다. 1968 년에는 결핵예방법을 제정하여 본격적으로 정부주도하의 결핵관리정책이 시행되기 시작하였으며, 2006 년에는 결핵퇴치 2030 계획을 수립하였고, 2013 년부터는 5 년마다 국가 결핵관리 종합계획을 수립하여 다양한 결핵관리정책을 시행해 오고 있다. 특히, 2011 년에는 결핵 공공·민간협력 모델(PPM, TB Public-Private Mix Model)을 구축하여, 전국 252 개 보건소 및 120 여개 의료기관에 결핵 관리 전담 간호사를 배치하여 결핵 환자를 집중적으로 관리해 오고 있다. 2004 년에는 결핵 접촉자 조사가 시작되었는데, 2009 년에는 결핵 접촉자 조사 관련 예산을 편성하였고, 2013 년부터는 중앙 역학조사반을 운영하면서 본격적인 결핵 접촉자 조사를 시행하기 시작하였다. 또한 2011 년부터는 결핵 진단 및 치료를 국가에서 무료로 제공하고 있으며, 2017 년에는 약 120 만 명에 대한 잠복결핵감염 검진 및 치료를 실시한 바 있으며, 2018 년에는 노인 결핵검진 시범사업과 외국인 결핵 및 잠복결핵검진 시범사업을 실시하는 등 그간 사각지대에

있던 취약계층에 대한 프로그램을 강화할 계획에 있다(그림 1). 한국의 결핵관리 틀은 그림 2 와 같다. 결핵예방법과 결핵관리종합계획, 연구개발 등이 국가결핵관리정책의 기초를 이루고 있으며, 예방, 조기발견, 환자치료·관리의 영역에서 다양한 정책 및 프로그램들이 운용되고 있다(그림 2).

결핵 예산 (TB Budget)

국가결핵관리 예산은 국비 기준으로 2007 년에 99 억원, 2010 년 149 억원에서 2011 년 447 억원으로 급격히 증가하였는데, 이는 2011 년부터 건강보험 결핵 진료비의 10%에 해당하는 본인부담에 대한 국고 지원이 시작되었기 때문이다. 이처럼 국고에서 지원하던 건강보험의 결핵 치료비 본인부담 분은 2017 년에 전면 면제되어, 전액 건강보험 재정으로 지출됨에 따라, 2017 년에는 관련 예산을 결핵예방법에 따른 의료인, 유치원 교사, 어린이집 및 아동복지시설 종사자 등에 대한 잠복결핵감염 검진사업비 89 억 원으로 편성 및 지출하여 결핵 및 잠복결핵감염 조기발견사업비의 증가 폭이 컸다. 그러나 잠복결핵감염검진 및 치료는 법 개정에 따른 2017 년 단년도 사업으로, 2018 년에는 의료인에 대한 잠복결핵감염검진 및 치료비 일부만 반영되었고, 노인결핵검진사업 6.2 억 원 및 외국인 결핵· 잠복결핵검진사업 4.5 억 원 등 신규 사업 예산이 반영되었음에도 불구하고 2018 년 전체 예산은 343 억 원 수준에 머물렀다(표 1).

결핵 예방 (TB Prevention)

한국의 높은 결핵 발생률에도 불구하고 국민들의 결핵에 대한 이해도와 관심은 매우 낮은 실정이다. 이에 정부에서는 결핵에 대한 인식 향상, 결핵 조기검진 유도, 올바른 기침예절 등을 위해 다양한 홍보를 실시하고 있다. 한국에서는 결핵 예방접종이 의무화되어 있고 국가가 예산을 지원하는데, 결핵 예방접종률은 1965 년 16.4%에서 1990 년 65.7%로 증가하였고, 2003 년 87.0%, 2013 년에는 99.8%로 증가하였다[6,7]. 한국은 결핵 예방백신(BCG 백신)을 전량 수입에

의존하고 있는데, 때때로 발생하는 결핵 예방백신의 수급 불안정을 해소하고 적기에 백신을 공급하기 위해서 2020 년을 목표로 2014 년부터 결핵 예방백신(BCG 백신)을 자체 개발하고 있다.

한편, 한국의 잠복결핵감염율은 33%로 매우 높은 수준이다[3,8]. 이에 한국에서는 고위험군에 대한 잠복결핵감염 진단 및 치료를 통해 예방을 도모하고 있다. 세계보건기구[9]가 체계적인 잠복결핵감염 진단과 치료를 시행하도록 강하게 권고하고 있는 HIV 감염인, 접촉자, 장기이식자, TNF 사용자, 투석 환자, 규폐증 등에 대해서는 잠복결핵감염 검사가 건강보험의 요양급여로 제공되고 있다[3,10]. 또한 2017 년부터는 결핵예방법에 따라 의료기관 종사자, 산후조리원 종사자, 어린이집 종사자, 유치원, 초·중·고등학교 교사 등은 결핵 및 잠복결핵감염 검진이 의무화되었고, 2017 년에 이들 중 120 만 여명을 대상으로 체계적인 잠복결핵감염 검진과 치료 비용을 국가에서 제공한 바 있다[3,10].

결핵 조기발견 (Early Detection of TB)

한국에서는 중학교 및 고등학교 입학생을 대상으로 결핵검진(흉부 X-ray)을 학생 건강검진에 포함하여 실시하고 있고, 성인의 경우는 건강보험가입자에 대해 1~2 년 마다 결핵검진을 포함하여 건강검진을 실시하고 있다. 또한 결핵예방법에 따라 의료인, 교사 등은 의무적으로 주기적인 결핵 및 잠복결핵감염 검진을 의무적으로 실시하도록 하고 있다. 그러나 노인, 외국인, 노숙인 등의 취약계층은 결핵검진의 사각지대에 놓여 있다. 이에 정부는 2018 년부터 노인 결핵검진과 외국인 대상 결핵 및 잠복결핵검진 시범사업을 추진하여 향후 이들에 대한 체계적인 조기발견 전략을 마련할 계획이다.

한편, 결핵 조기발견의 다른 중요한 한 축은 결핵 접촉자 조사(TB contact investigation)인데, 한국은 결핵예방법에 따라 가족 접촉자 및 집단시설에서의 접촉자를 대상으로 결핵 접촉자 조사를 실시하고 있다. 2004 년 13 건의 결핵 접촉자 조사로 시작하여, 이를 본격적으로 시작한 2013 년에는 1,142 건으로 증가하였고, 2014 년 1,405 건, 2015 년 2,639 건, 2016 년 3,502 건으로 지속적인

Volume: 40, Article ID: e2018036
https://doi.org/10.4178/epih.e2018036
증가추세를 보이고 있다. 2016 년 결핵 접촉자 조사 대상은 146,911 명이었고, 잠복결핵감염자로 진단된 경우는 12,707 명이었다[11].

결핵 치료 및 관리 (TB Treatment and Management)

한국에서 결핵 환자는 결핵예방법에 따라 전수 신고·등록하도록 되어 있고, 진단과 치료 비용을 국가에서 무료로 지원함과 동시에 완치에 이를 때까지 관리를 하고 있다. 또한 국가 결핵관리 프로그램에서 잠복결핵감염 검진과 치료에 대해서도 무료로 비용을 지원하고 있다. 한국에서 전염성 결핵 환자로 확인되면 이들 중 근로자 또는 학생의 경우 전염성 소실이 확인될 때까지 업무종사 및 등교 일시 제한과 더불어 최소 2주간의 복약 확인을 시행하고 있다. 다만, 한국에서는 2013년부터 직접 복약 확인 치료 서비스(DOT, Directly Observed Treatment)를 권고하고 있는데, 세계보건기구(WHO)가 제시하는 가이드라인에는 못 미치고 있는 실정이다. 또한 치료 비순응 환자를 대상으로 전화상담, 가정방문 등을 통해 치료를 관리하고 있으며, 이러한 비순응 결핵환자 관리 성공률은 2014년 56.4%에서 2015년 50.0%, 2016년 63.1%로 개선되었다. 2016년 전체 결핵 환자 치료성공률은 84.3%이며, 결핵 신환자 치료성공률은 86.7%, 도말양성 결핵신환자 치료성공률은 83.3%로 나타났으며, 이러한 지표들은 매년 조금씩이나마 향상되고 있다[3].

한국의 리팜핀(RR, Rifampicin—Resistance) 및 다제내성(MDR, Multi-Drug Resistance) 결핵환자(RR/MDR-TB)는 신환자 기준으로 3.4%인데 이는 경제협력개발기구(OECD) 가입국 평균 2.6%보다 높고, 재치료자 기준으로는 11%로 OECD 가입국 평균 12%보다는 다소 낮은 편이다[12]. 특히, 한국에서 다제내성 결핵환자 중 외국인의 비율은 2011년 4.5%에서 2016년에 20.5%로 증가하였다. 한국은 다제내성결핵환자 치료성공률을 높이고자 최근에는 고가의 다제내성 결핵 신약을 건강보험에 포함시켜 무료로 제공하고 있다. 한국의 리팜핀 및 다제내성 결핵환자(RR/MDR-TB) 치료성공률은 63%로 OECD 가입국 평균 62%와 비슷한 수준이다[12].

한편, 한국에서 결핵환자 치료 및 관리에 있어서 매우 성공적인 모델 중 하나로 꼽을 수 있는 것은 결핵 공공·민간 협력 모델(TB public-private mix(PPM) models)이다. 2007 년

https://doi.org/10.4178/epih.e2018036
시범사업을 거쳐 2011년 전국으로 확대된 이 모델은 전국 252개 보건소와 120여개 종합병원간 유기적으로 협력하여 결핵 환자의 치료 성공률을 높이는데 기여하였는데, 보건소와 병원에 420 명의 결핵 관리 전담 간호사를 배치하여 결핵 환자 상담, 복약지도, 비순응 환자 관리 등 체계적인 결핵관리를 실시하고 있다.

고찰 (DISCUSSION)

한국의 결핵 발생과 사망은 1950 년 한국 전쟁 이후 최고점을 보인 이후 급속도로 감소해 왔으나(그림 1), 여전히 경제협력개발기구(OECD) 가입국 중에서 가장 높은 수준을 보이고 있다[1,2]. 한국의 높은 경제 수준에도 불구하고 결핵 발생률이 높은 이유는 한국 전쟁을 꼽을 수 있다. 전쟁 이후 결핵의 유행은 다른 나라의 사례에서도 나타나는 바[13,14], 한국은 한국 전쟁을 겪으면서 낮은 영양 상태, 밀집된 주거 환경, 낮은 의료 접근성 등 열악한 보건 환경 속에서 이 당시 국민의 상당수가 잠복결핵에 감염된 것으로 추정된다[3,5]. 한국의 잠복결핵감염율은 1960 년 64.2%, 1975 년 59.3%, 1990 년 44.4%, 2016 년 33.2%로 꾸준히 감소하고 있지만, 미국 등의 선진국에 여전히 매우 높은 수준이다[3,8,15]. 이처럼 높은 잠복결핵감염율은 한국의 결핵감소에 있어서 가장 큰 장애 요인 중 하나라고 할 수 있겠다. 2017 년 한국 정부는 결핵예방법 개정을 통해 의료인, 산후조리업 종사자, 교사 등에 대해 결핵 및 잠복결핵감염 검진을 의무화하고, 120 만 명에 대한 체계적인 잠복결핵감염 검진 및 치료를 실시한 바 있다[3,10].

최근 한국에서는 결핵 환자의 신속한 진단과 치료에 따라 치료성공률의 개선이 이루어졌고, 여기에는 민간·공공 협력 결핵 관리 사업(PPM)을 통한 환자 치료 및 관리체계가 상당 부분을 기여함으로써 결핵 발생률이 지속적으로 감소하고 있다[3]. 또한 최근 다른 연령층보다 20 세 미만의 연령층에서 결핵 환자수가 급감하였는데, 이는 결핵 발생률이 점차 낮아지고 있는 시기에 출생한 세대라는 점과 더불어 학교에서의 결핵검진과 결핵 접촉자 조사의 효과로 유추해 볼 수 있겠다[3,4]. 한편, 한국에서 전체 결핵 환자 중 65 세 이상 노인의 비율이 42%를 차지하고, 결핵

사망자 중 82%가 노인인데, 노인의 결핵 검진 수검율은 다른 연령층에 비해 낮을 뿐 아니라, 노인 대상 결핵관리 프로그램도 매우 부족한 실정이다. 이러한 문제를 해결하기 위해 정부는 2018 년부터 노인 결핵검진 및 조기발견 시범사업을 실시하여 노인 대상의 결핵관리 프로그램을 마련할 계획에 있다. 다른 한편, 한국에서는 결핵 발생률이 높은 19 개 국가에서 거주 및 취업 목적으로 입국하는 외국인에 대해 결핵검진이 의무화되어 있으나, 국내에 입국하여 체류하는 동안 발생하는 결핵에 대한 관리는 어려운 실정이다. 따라서 국내에 체류하는 동안 주기적인 결핵검진과 더불어 잠복결핵감염 검진을 실시하는 방안을 검토하기 위해 2018 년에 시범사업을 실시할 계획이다.

한국 정부는 인구 10 만 명당 77 명인 결핵발생률을 2022 년까지 인구 10 만 명당 40 명까지 낮추는 것을 목표로 제 2 기 결핵관리종합계획(2018~2022)을 수립하였다. 정부는 향후 5 년 동안 이를 충실히 이행해 나가면서 우선순위에 따라 관련 예산 투입을 확대해 나가야 할 것이다. 먼저 한국의 결핵관리에서 가장 우선시 되어야 할 것은 결핵환자의 조기 진단과 치료일 것이다. 결핵의 신속한 진단과 치료율 제고를 위해 진단 및 치료 관련 건강보험 적용이 확대되어야 하며, 일선 의료기관에서 결핵 환자의 신속한 진단과 표준 치료가 이루어질 수 있도록 의료인 대상 교육과 결핵 환자를 집중 관리하는 민간·공공 협력 결핵 관리 사업(PPM)의 내실화가 필요하다. 또한 다제내성 결핵환자의 치료기간 단축 및 치료성공률 제고를 위한 연구개발이 확대될 필요가 있다. 다른 한편, 그간 미진했던 노인, 외국인, 노숙인 등 취약계층에 대한 결핵 조기발견과 체계적인 치료 방안도 모색될 필요가 있다. 취약계층 결핵 치료에 있어서는 지역사회 중심의 보건복지 통합 환자관리 모형을 개발·도입하고, 결핵 격리병상과 결핵 관리 전담 간호사 배치 확대 등의 자원 확대도 요구된다. 둘째, 결핵 접촉자 조사를 보다 강화하여 학교, 병원, 교도소 등 집단시설별로 최적화된 접촉자 조사 체계를 갖출 필요가 있다. 결핵 접촉자 조사는 일상 접촉자(casual contacts)보다는 밀접 접촉자(close contacts)에 우선순위에 두고, 조사대상자의 수검률을 높이고, 잠복결핵감염 양성자에 대해서는 치료율을 높이기 위한 노력을 기울일 필요가 있다[4,12,16-20]. 셋째, 결핵 예방 차원에서는 주기적인 결핵검진을 받을 수 있도록 대상자별 홍보 전략을 마련하고, 반복적으로 수급난을 겪고 있는 결핵 예방백신(BCG 백신)의 개발을 성공적으로 완료하여 적기에 백신을 공급할 수 있도록 해야 할 것이다. 또한 의료인 등 고위험군 대상으로 한정하여 잠복결핵감염 검진 및 치료를 지속적으로

실시할 필요가 있다. 2017 년에 120 만 명을 대상으로 실시한 잠복결핵감염 검진에 대해서는 장기적으로 추적·관리하여 잠복결핵감염 검진과 치료의 효과에 대한 평가 또한 요구된다 하겠다[12].

이해관계 (CONFLICT OF INTEREST)

연구자는 본 연구와 관련하여 어떠한 이해관계도 없음을 밝혀둔다.

Volume: 40, Article ID: e2018036
https://doi.org/10.4178/epih.e2018036

참고문헌 (REFERENCES)

1. WHO. 2016 Global Tuberculosis Report. Geneva: Word Health Organization; 2017.
2. KCDC. 2017 Annual Report on the Notified Tuberculosis in Korea. Osong: Korea Centers for Diseases Control and Prevention; 2018.
3. Cho KS. Tuberculosis Control in the Republic of Korea. Health and Social Welfare Review 2017;37(4):179-212.
4. Cho KS. Tuberculosis incidence and latent tuberculosis infection prevalence in high schools in Korea. KCDC PHWR 2018;11(6):152-58.
5. Kim J. H. & Yim J. J. Achievements in and Challenges of Tuberculosis Control in South Korea. Emerging Infectious Diseases 2015;21(11):1913-1920.
6. KMOHW, KNTA. The 7th National Tuberculosis Prevalence Survey. Gwacheon: Korea Ministry of Health and Welfare, Korean National Tuberculosis Association; 1996 (Korean).
7. Lee SG, Jeon SY. 2013 National Immunization Survey. Osong: Korea Centers for Diseases Control and Prevention;2013 (Korean)
8. KCDC, KIT. 7th Korea National Health and Nutrition Examination Survey 1st year (2016) Tuberculin Survey Support and Quality Control. Osong: Korea Centers for Diseases Control and Prevention, Korean Institute of Tuberculosis; 2016. (Korean)
9. WHO. (2015c). *Guidelines on the management of latent tuberculosis infection*. Geneva: Word Health Organization.
10. Cho KS, Park WS, Jeong HR, Kim MJ, Park SJ, Park AY, et al. Prevalence of latent tuberculosis infection at congregated settings in the Republic of Korea, 2017. KCDC PHWR 2018;11(12):348-354.
11. KCDC. 2016 Report on the Contact Investigations at Congregated Settings. Osong: Korea Centers for Diseases Control and Prevention;2017 (Korean).
12. Cho KS, Kim NH. Multidrug-resistant tuberculosis status and performance of the healthcare review committee for new drugs for MDR-TB in the Republic of Korea. KCDC PHWR 2017;10(50):1362-1368.

13. Drolet GJ. World War I and tuberculosis: a statistical summary and review. Am J Public Health Nations Health 1945;35:689–697. http://dx.doi.org/10.2105/AJPH.35.7.689

14. Byerly CR. Chapter 8. Camp Follower: Tuberculosis in World War II in Good Tuberculosis Men: The Army Medical Department’s Struggle with Tuberculosis, Texas: Fort Sam Houston, Texas Borden Institute, United States Army Medical Department Center and School; 2013.

15. Horsburgh CR, Rubin EJ. Latent Tuberculosis Infection in the United States. N Engl J Med 2011;364:1441-1448.

16. Grzybowski S, Barnett GD, Styblo K. Contacts of cases of active pulmonary tuberculosis. Bull Int Union Tunerc 1975;50:90-106.

17. Reichler MR, Reves R, Bur S, et al. Evaluation of investigations conducted to detect and prevent transmission of tuberculosis JAMA 2002;287:991-995.

18. Fox GJ, Barry SE, Britton WJ, Marks GB. Contact investigation for tuberculosis: a systematic review and meta-analysis. Eur Respir 2013;41(1):140-156.

19. Sandgren A, Noordegraaf-Schouten MV, Kessel F, Stuurman A, Oordt-Speets A, Werf MJ. Initiation and completion rates for latent tuberculosis infection treatment. *B*MC Infectious Diseases 2016; 16:204.

20. Zenner D, Loutet MG, Harris R, Wilson S, Ormerod LP. Evaluating 17 years of latent tuberculosis infection screening in north-west England: a retrospective cohort study of reactivation. European Respiratory Journal 2017;50:1602505. doi:10.1183/13993003.02505-2016.

Volume: 40, Article ID: e2018036
https://doi.org/10.4178/epih.e2018036

Table 1. TB budgets of central government by year

unit: billion KR won[1]

Category	2007	2008	2009	2010	2011	2012	2013	2014	2015	2016	2017	2018
TB prevention and Infrastructure	4.4	4.1	1.6	1.9	1.9	2.3	2.7	2.3	2.5	2.6	2.6	2.7
TB treatment and management	0.2	0.2	5.5	6.6	33.6	18.6	18.5	19.6	20.5	19.7	12.7	13.1
TB and LTBI screening	-	-	0.6	0.6	8.0	10.3	12.2	11.0	10.6	12.9	20.8	14.1
R&D and others	5.3	7.7	4.7	5.8	1.2	7.9	5.7	3.6	3.4	4.1	5.1	4.4
Total	9.9	12.0	12.4	14.9	44.7	39.1	39.1	36.5	37.0	39.3	41.2	34.3

[1]1000 KR won = 1 US dollar
Source: Cho KS. Tuberculosis Control in the Republic of Korea. Health and Social Welfare Review 2017;37(4):179-212.

Volume: 40, Article ID: e2018036
https://doi.org/10.4178/epih.e2018036

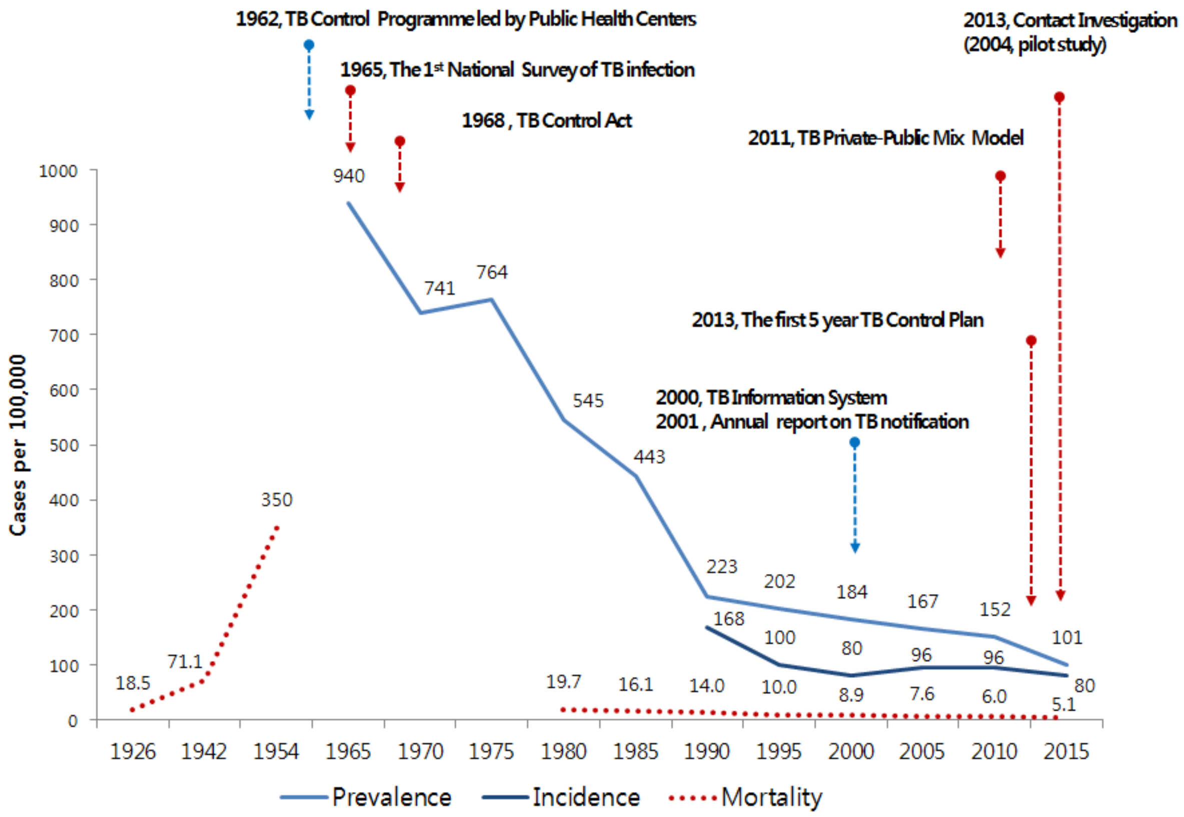

Source: Cho KS. Tuberculosis Control in the Republic of Korea. Health and Social Welfare Review 2017;37(4):179-212.

Figure 1. TB prevalence, incidence and mortality by year

Volume: 40, Article ID: e2018036
https://doi.org/10.4178/epih.e2018036

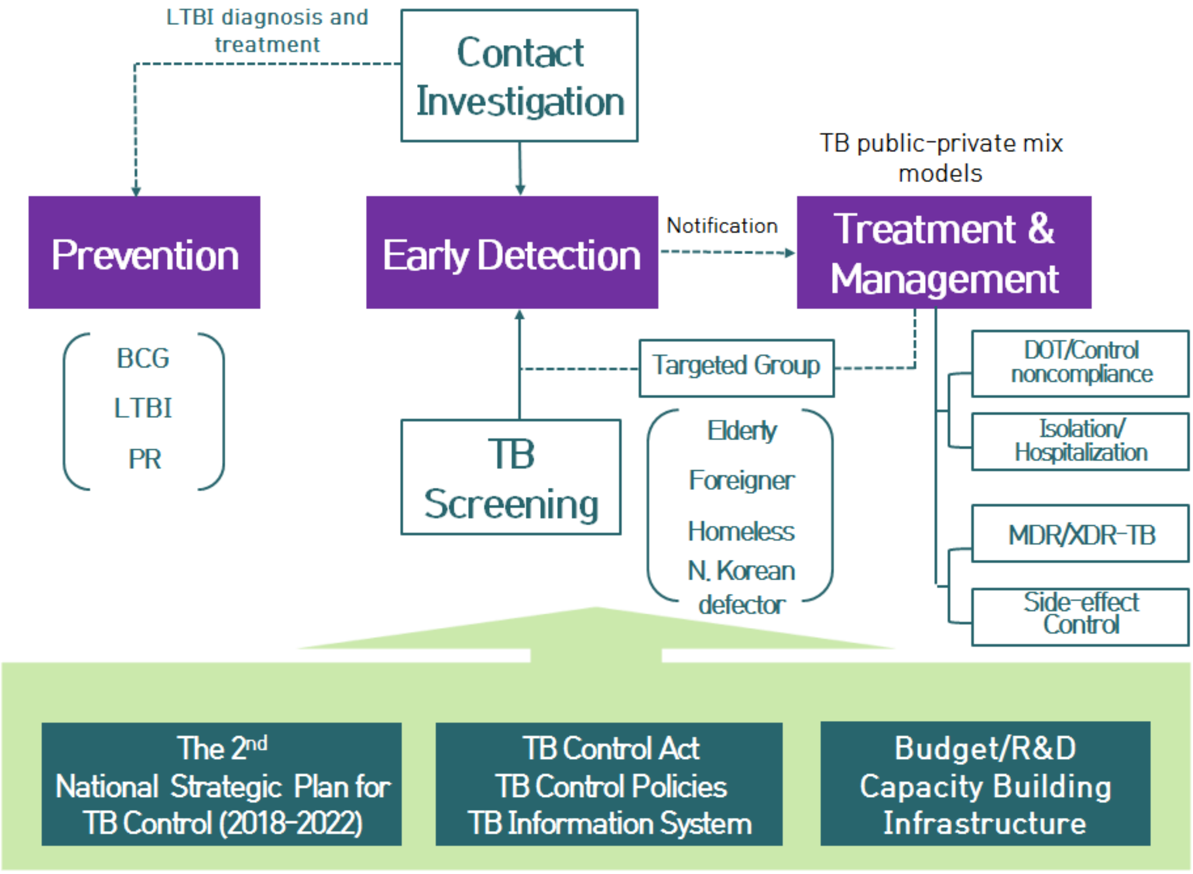

Source: Cho KS. Tuberculosis Control in the Republic of Korea. Health and Social Welfare Review 2017;37(4):179-212.

Figure 2. TB Control Framework in the Republic of Korea

Appendix 1. Comparison of TB prevalence and incidence by sources of statistics

unit: case per 100,000

Year	Prevalence				Incidence			
	TB Survey[1)]		WHO[2)]	Notified TB[3)]	Research[4)]		Notified TB[3)]	WHO[5)]
	Radiologically active TB cases	Bacteriologically confirmed TB cases	Prevalence	Total TB cases notified	Radiologically active TB cases	Bacteriologically confirmed TB cases	New TB cases notified	Incidence
1965	5,065	940	-	-	-	-	-	-
1970	4,222	741	-	-	-	-	-	-
1975	3,326	764	-	-	-	-	-	-
1980	2,509	545	-	-	-	-	-	-
1985	2,158	443	-	-	393 [2]	83 [2]	-	-
1990	1,842	241	223	-	202 [3]	81 [3]	-	168
1995	1,032	219	202	-	-	-	-	100
2000	-	-	184	96.3	-	-	71.3	80
2005	-	-	167	96.5	-	-	72.4	96
2010	-	-	152	96.4	-	-	70.8	96
2015	-	-	101 [1]	80.2	-	-	63.2	80

[1] year of 2014
[2] year of 1988-1990
[3] year of 1992-1994

Original source: 1) KMOHW, KNTA. The 7th National Tuberculosis Prevalence Survey. Gwacheon: Korea Ministry of Health and Welfare, Korean National Tuberculosis Association; 1996.
2) WHO. 2014 Global Tuberculosis Report. Geneva: WHO;2015
3) KCDC. 2016 Annual Report on the Notified Tuberculosis in Korea. Osong: Korea Centers for Diseases Control and Prevention; 2017.
4) Kim SJ, Hong YP, Lew WJ, Yang SC, Lee EG. Incidence of pulmonary tuberculosis in Korean civil servants. Tubercle and Lung Disease, 1995;76:534-539; Bai GH, Kim SJ, Lee EK, Lew WJ. Incidence of pulmonary tuberculosis in Korean civil servants: second study, 1992–1994. Int J Tuberc Lung Dis, 2001;5(4):346–353.
5)WHO. 2015 Global Tuberculosis Report. Geneva: WHO; 2016.

Source: Cho KS. Tuberculosis Control in the Republic of Korea. Health and Social Welfare Review 2017;37(4):179-212.

Volume: 40, Article ID: e2018036
https://doi.org/10.4178/epih.e2018036

Appendix 2. Rates of positive LTBI

unit: %, yrs

Category		Rate of positive LTBI (%)	Mean age of individuals tested	Mean age of positive LTBI
All ages[1),2),4)]	1965	64.2 [1]	-	-
	1970	61.3 [1]	-	-
	1975	59.3 [1]	-	-
	1990	44.4 [1]	-	-
	2016	33.2 [1]	40.7	-
Adolescents and young people[3)]	First grade high school students	2.1 [2]	15.3	15.4
	Out of school youth	3.2 [2]	16.7	20.0
	Military conscripts	2.9 [2]	-	-
Facility workers[3)]	Health care workers	17.5 [2]	37.9	45.7
	Nursery workers	19.3 [2]	40.6	45.8
	Workers in children welfare facilities	21.7 [2]	42.5	49.4
	Workers in other welfare facilities	28.5 [2]	48.6	53.7
	Workers in postnatal care centers	33.2 [2]	-	-
Teachers[3)]	Teachers in kindergarten	15.2 [2]	36.8	46.2
	Teachers in elementary/middle/high schools	18.0 [2]	43.1	48.4
Prisoners[3)]		34.0 [2]	-	-

[1] Results from tuberculin skin test(TST).
[2] Results from Interferon-gamma releasing assay(IGRA)

Source: 1) KMOHW, KNTA. The 7th National Tuberculosis Prevalence Survey. Gwacheon: Korea Ministry of Health and Welfare, Korean National Tuberculosis Association; 1996.
2) KCDC, KIT. 7th Korea National Health and Nutrition Examination Survey 1st year (2016) Tuberculin Survey Support and Quality Control. Osong: Korea Centers for Diseases Control and Prevention, Korean Institute of Tuberculosis; 2016.
3) Cho KS, Park WS, Jeong HR, Kim MJ, Park SJ, Park AY, et al. Prevalence of latent tuberculosis infection at congregated settings in the Republic of Korea, 2017. KCDC PHWR 2018;11(12):348-354.
4) Cho KS. Tuberculosis Control in the Republic of Korea. Health and Social Welfare Review 2017;37(4):179-212.
.

Volume: 40, Article ID: e2018036
https://doi.org/10.4178/epih.e2018036

Appendix 3. Age specific rates of positive LTBI by type of LTBI test

unit: %, yrs

Age group	Rate of positive LTBI by IGRA in 2017 [1,1)]	Rate of positive LTBI by TST in 2016 [2,2)]
10-19	n.a.	6.5
20-29	5.5	10.9
30-39	12.7	36.4
40-49	23.0	46.1
50-59	35.1	48.7
60-64	42.0	45.0
Total	20.3	33.2
Mean age of of individuals tested	41.0	40.7
Mean age of positive LTBI	47.9	n.a.

[1] Results from 543,538 facility workers including health care workers, nursery workers, and workers in children and other welfare facilities.

[2] Results from general population (n=2,051).

Source: 1) Cho KS, Park WS, Jeong HR, Kim MJ, Park SJ, Park AY, et al. Prevalence of latent tuberculosis infection at congregated settings in the Republic of Korea, 2017. KCDC PHWR 2018;11(12):348-354.

2) KCDC, KIT. 7th Korea National Health and Nutrition Examination Survey 1st year (2016) Tuberculin Survey Support and Quality Control. Osong: Korea Centers for Diseases Control and Prevention, Korean Institute of Tuberculosis; 2016.

Volume: 40, Article ID: e2018036
https://doi.org/10.4178/epih.e2018036

Appendix 4. Number of TB contact investigations, 2013-2016

unit: case

Category	2013	2014	2015	2016
School	750	758	708	659
Healthcare facility	81	202	549	856
Army/Police	108	135	112	128
Social welfare facility	120	160	324	528
Prison	27	29	33	46
Work place	31	91	845	1,167
Others	25	30	68	118
Total	1,142	1,405	2,639	3,502

Source: 1) KCDC. 2016 Report on the Contact Investigations at Congregated Settings. Osong: Korea Centers for Diseases Control and Prevention;2017.
2) Cho KS. Tuberculosis Control in the Republic of Korea. Health and Social Welfare Review 2017;37(4):179-212.
