# Supplementary material for: Tuberculosis control in the Republic of Korea
Source: Epidemiol Health. 2018 Aug 2;40:e2018036. doi: 10.4178/epih.e2018036 (PMC6335497; doi:10.4178/epih.e2018036)
Supplement: Supplementary file 2 [file epih-40-e2018036-supplementary1.pdf]

Supplementary Material 1

Table S1. Comparison of TB prevalence and incidence by sources of statistics

unit: case per 100,000

Year	Prevalence				Incidence			
	TB Survey[1)]		WHO[2)]	Notified TB[3)]	Research[4)]		Notified TB[3)]	WHO[5)]
	Radiologically active TB cases	Bacteriologically confirmed TB cases	Prevalence	Total TB cases notified	Radiologically active TB cases	Bacteriologically confirmed TB cases	New TB cases notified	Incidence
1965	5,065	940	-	-	-	-	-	-
1970	4,222	741	-	-	-	-	-	-
1975	3,326	764	-	-	-	-	-	-
1980	2,509	545	-	-	-	-	-	-
1985	2,158	443	-	-	393**	83**	-	-
1990	1,842	241	223	-	202***	81***	-	168
1995	1,032	219	202	-	-	-	-	100
2000	-	-	184	96.3	-	-	71.3	80
2005	-	-	167	96.5	-	-	72.4	96
2010	-	-	152	96.4	-	-	70.8	96
2015	-	-	101*	80.2	-	-	63.2	80

note: * year of 2014, ** year of 1988-1990, *** year of 1992-1994

Original source: 1) KMOHW, KNTA. The 7th National Tuberculosis Prevalence Survey. Gwacheon: Korea Ministry of Health and Welfare, Korean National Tuberculosis Association; 1996.

2) WHO. 2014 Global Tuberculosis Report. Geneva: WHO;2015.

3) KCDC. 2016 Annual Report on the Notified Tuberculosis in Korea. Osong: Korea Centers for Diseases Control and Prevention; 2017.

4) Kim SJ, Hong YP, Lew WJ, Yang SC, Lee EG. Incidence of pulmonary tuberculosis in Korean civil servants. Tubercle and Lung Disease, 1995;76:534-539; Bai GH, Kim SJ, Lee EK, Lew WJ. Incidence of pulmonary tuberculosis in Korean civil servants: second study, 1992–1994. Int J Tuberc Lung Dis, 2001;5(4):346–353.

5)WHO. 2015 Global Tuberculosis Report. Geneva: WHO; 2016.

Source: Cho KS. Tuberculosis Control in the Republic of Korea. Health and Social Welfare Review 2017;37(4):179-212.
