# Supplementary material for: Tuberculosis control in the Republic of Korea
Source: Epidemiol Health. 2018 Aug 2;40:e2018036. doi: 10.4178/epih.e2018036 (PMC6335497; doi:10.4178/epih.e2018036)
Supplement: Supplementary file 3 [file epih-40-e2018036-supplementary2.pdf]

Supplementary Material 2

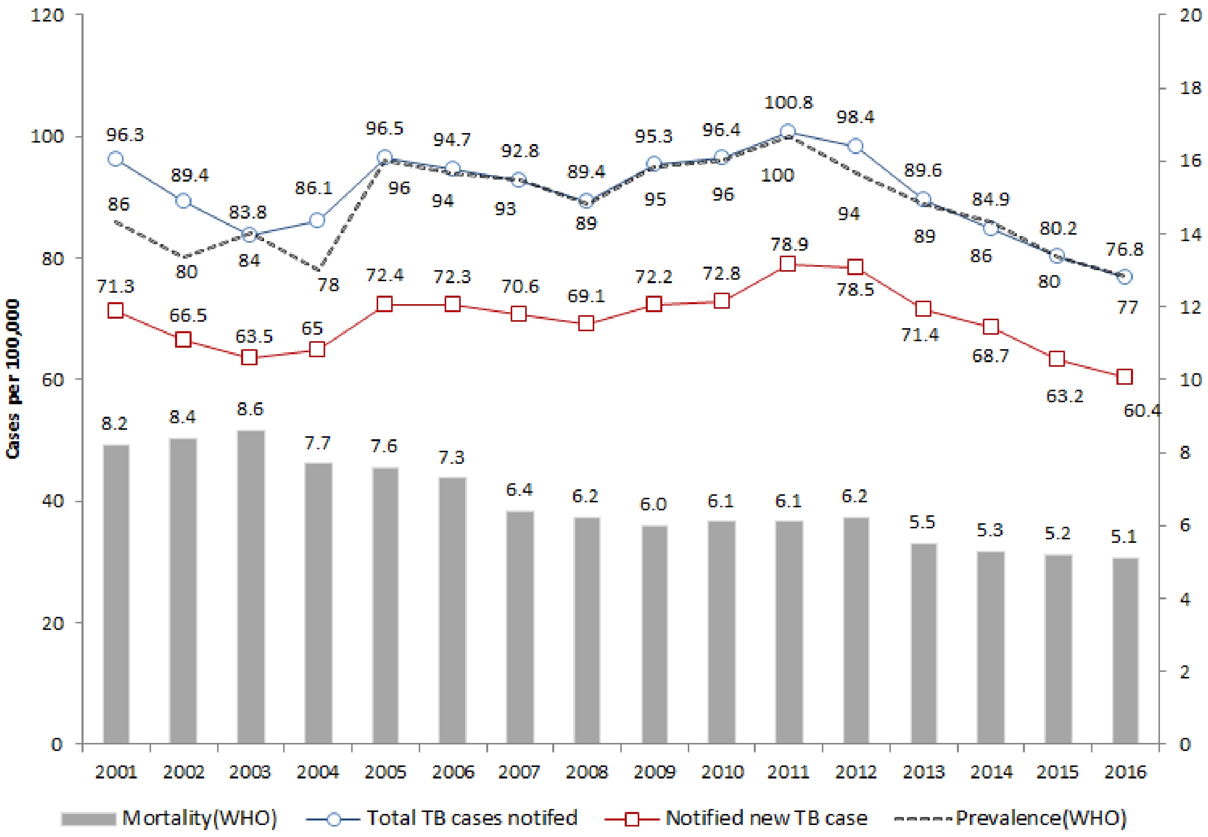

Source: 1) KCDC. 2016 Annual Report on the Notified Tuberculosis in Korea. Osong: Korea Centers for Diseases Control and Prevention; 2017.

2) WHO. 2015 Global Tuberculosis Report. Geneva: WHO; 2016; WHO. 2016 Global Tuberculosis Report. Geneva: WHO; 2017

3) Cho KS. Tuberculosis Control in the Republic of Korea. Health and Social Welfare Review 2017;37(4):179-212.

Figure S1. TB prevalence, mortality, and TB notification rate by year.
