# Supplementary material for: Tuberculosis control in the Republic of Korea
Source: Epidemiol Health. 2018 Aug 2;40:e2018036. doi: 10.4178/epih.e2018036 (PMC6335497; doi:10.4178/epih.e2018036)
Supplement: Supplementary file 4 [file epih-40-e2018036-supplementary3.pdf]

Supplementary Material 3

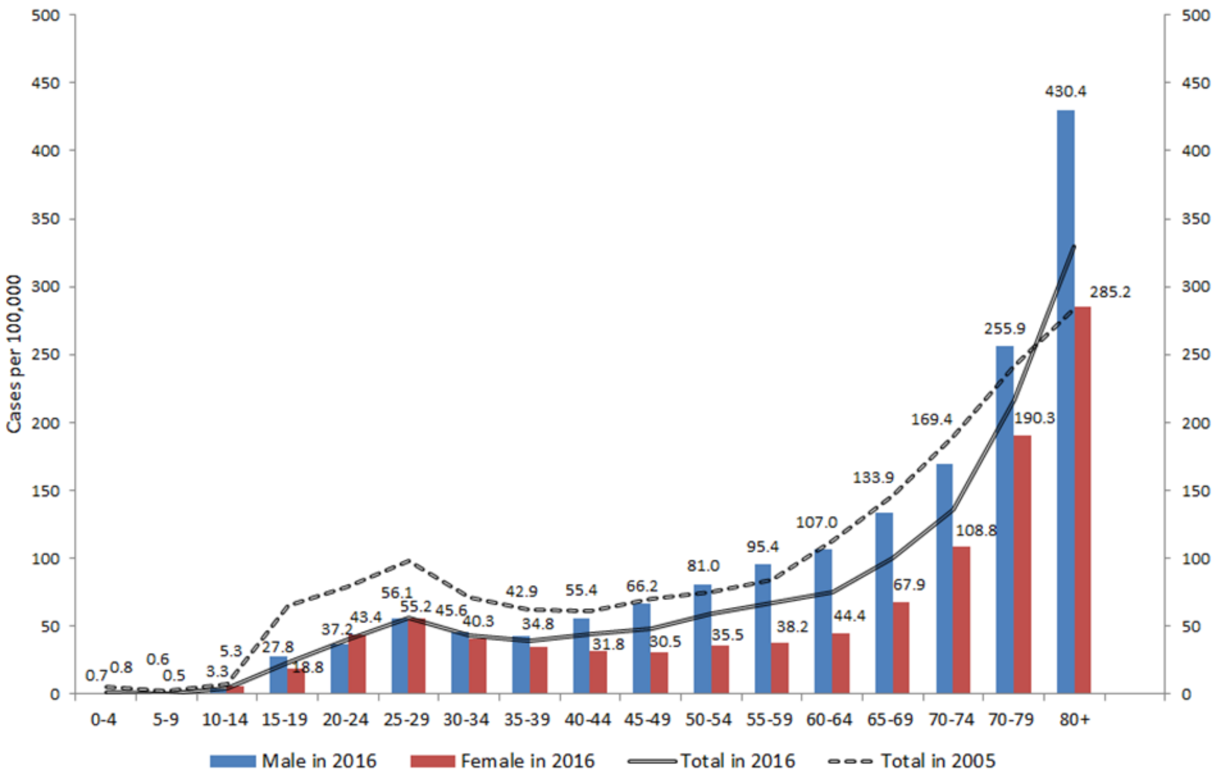

Source: 1) KCDC. 2016 Annual Report on the Notified Tuberculosis in Korea. Osong: Korea Centers for Diseases Control and Prevention; 2017.

2) Cho KS. Tuberculosis Control in the Republic of Korea. Health and Social Welfare Review 2017;37(4):179-212.

Figure S2. Age-gender specific new TB notification rate in 2016.
