# Supplementary material for: Tuberculosis control in the Republic of Korea
Source: Epidemiol Health. 2018 Aug 2;40:e2018036. doi: 10.4178/epih.e2018036 (PMC6335497; doi:10.4178/epih.e2018036)
Supplement: Supplementary file 5 [file epih-40-e2018036-supplementary4.pdf]

Supplementary Material 4

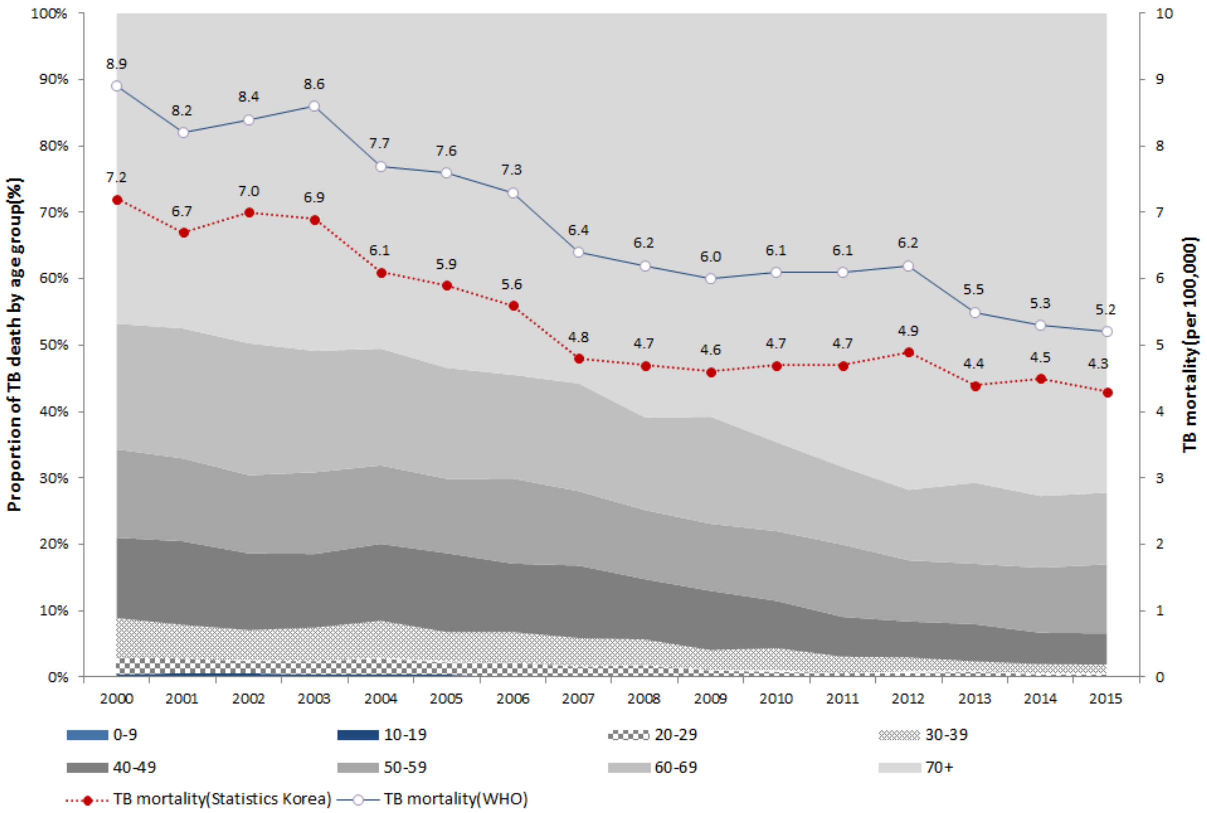

Source: 1) WHO. 2015 Global Tuberculosis Report. Geneva: WHO; 2016.

2) Cho KS. Tuberculosis Control in the Republic of Korea. Health and Social Welfare Review 2017;37(4):179-212.

Figure S3. TB mortality and proportion of TB deaths by year.
