# Supplementary material for: Tuberculosis control in the Republic of Korea
Source: Epidemiol Health. 2018 Aug 2;40:e2018036. doi: 10.4178/epih.e2018036 (PMC6335497; doi:10.4178/epih.e2018036)
Supplement: Supplementary file 6 [file epih-40-e2018036-supplementary5.pdf]

Supplementary Material 5

Table S2. Rates of positive LTBI by characteristics

unit: %, yrs

Category		Rate of positive LTBI (%)	Mean age	
			of individuals tested	of positive LTBI
All ages[1),2),4)*]	1965	64.2	-	-
	1970	61.3	-	-
	1975	59.3	-	-
	1990	44.4	-	-
	2016	33.2	40.7	-
Adolescents and young people[3)]	First grade high school students	2.1	15.3	15.4
	Out of school youth	3.2	16.7	20.0
	Military conscripts	2.9	-	-
Facility workers[3)]	Health care workers	17.5	37.9	45.7
	Nursery workers	19.3	40.6	45.8
	Workers in children welfare facilities	21.7	42.5	49.4
	Workers in other welfare facilities	28.5	48.6	53.7
	Workers in postnatal care centers	33.2	-	-
Teachers[3)]	Teachers in kindergarten	15.2	36.8	46.2
	Teachers in elementary/middle/high schools	18.0	43.1	48.4
Prisoners[3)]		34.0	-	-

note: Results from Interferon-gamma releasing assay(IGRA), * results from tuberculin skin test(TST).

Source:1) KMOHW, KNTA. The 7th National Tuberculosis Prevalence Survey. Gwacheon: Korea Ministry of Health and Welfare, Korean National Tuberculosis Association; 1996.

2)KCDC, KIT. 7th Korea National Health and Nutrition Examination Survey 1st year (2016) Tuberculin Survey Support and Quality Control. Osong: Korea Centers for Diseases Control and Prevention, Korean Institute of Tuberculosis; 2016.

3) Cho KS, Park WS, Jeong HR, Kim MJ, Park SJ, Park AY, et al. Prevalence of latent tuberculosis infection at congregated settings in the Republic of Korea, 2017. KCDC PHWR 2018;11(12):348-354.

4) Cho KS. Tuberculosis Control in the Republic of Korea. Health and Social Welfare Review 2017;37(4):179-212.
