# Supplementary material for: Tuberculosis control in the Republic of Korea
Source: Epidemiol Health. 2018 Aug 2;40:e2018036. doi: 10.4178/epih.e2018036 (PMC6335497; doi:10.4178/epih.e2018036)
Supplement: Supplementary file 7 [file epih-40-e2018036-supplementary6.pdf]

Supplementary Material 6

Table S3. Number of TB contact investigations for congregated settings, 2013-2016

unit: case

Category	2013	2014	2015	2016
School	750	758	708	659
Healthcare facility	81	202	549	856
Army/Police	108	135	112	128
Social welfare facility	120	160	324	528
Prison	27	29	33	46
Work place	31	91	845	1,167
Others	25	30	68	118
Total	1,142	1,405	2,639	3,502

Source: 1) KCDC. 2016 Report on the Contact Investigations at Congregated Settings. Osong: Korea Centers for Diseases Control and Prevention;2017.

2) Cho KS. Tuberculosis Control in the Republic of Korea. Health and Social Welfare Review 2017;37(4):179-212.
