# Supplementary material for: Tuberculosis control in the Republic of Korea
Source: Epidemiol Health. 2018 Aug 2;40:e2018036. doi: 10.4178/epih.e2018036 (PMC6335497; doi:10.4178/epih.e2018036)
Supplement: Supplementary file 8 [file epih-40-e2018036-supplementary7.pdf]

Supplementary Material 7

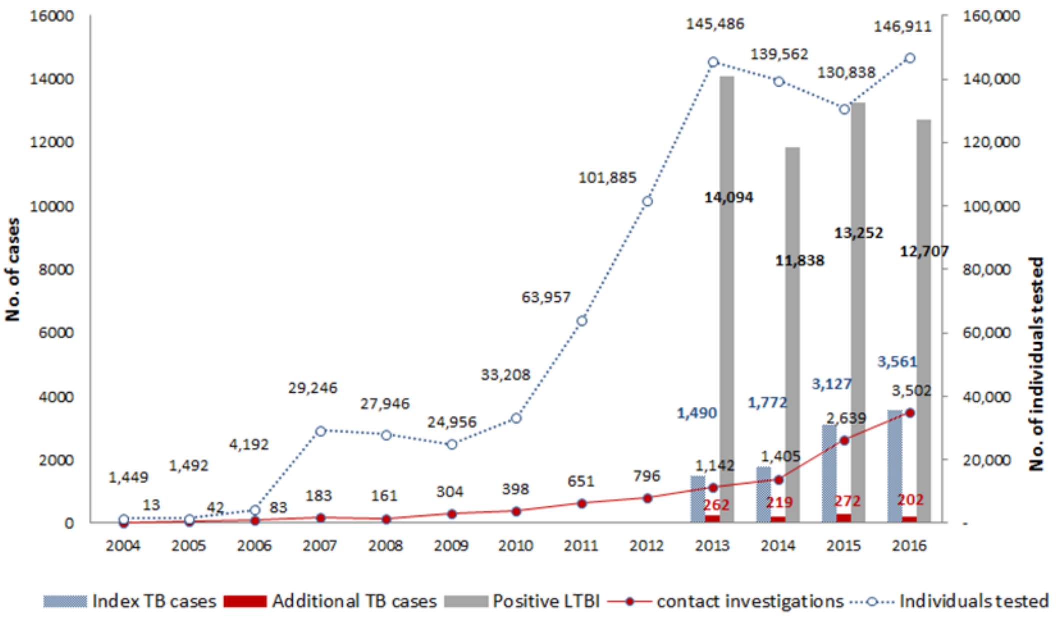

Source: Cho KS. Tuberculosis Control in the Republic of Korea. Health and Social Welfare Review 2017;37(4):179-212.

Figure S4. Number of TB contact investigations, index cases, detected cases, LTBI tests, and positive LTBI by year.
