# Supplementary material for: Tuberculosis control in the Republic of Korea
Source: Epidemiol Health. 2018 Aug 2;40:e2018036. doi: 10.4178/epih.e2018036 (PMC6335497; doi:10.4178/epih.e2018036)
Supplement: Supplementary file 9 [file epih-40-e2018036-supplementary8.pdf]

Supplementary Material 8

Table S4. Number of notified MDR-TB cases and treatment success rates by year

unit: case, %

Year		2011	2012	2013	2014	2015	2016
MDR-TB case (% of notified new TB cases)		975 (2.4)	1,212 (3.1)	951 (2.6)	856 (2.5)	787 (2.4)	852 (2.8)
Foreigner		44 (4.5)	90 (7.4)	89 (9.4)	101 (11.8)	129 (16.4)	175 (20.5)
	Chinese	11 (25.0)	26 (28.9)	63 (70.8)	79 (78.2)	101 (78.3)	143 (81.7)
	Others	33 (75.0)	64 (71.1)	26 (29.2)	20 (21.8)	28 (21.7)	32 (18.3)
Treatment success rate (%)		-	-	-	54.1	59.0	63.0

Source: 1) Cho KS. Tuberculosis Control in the Republic of Korea. Health and Social Welfare Review 2017;37(4):179-212.

2) Cho KS, Kim NH. Multidrug-resistant tuberculosis status and performance of the healthcare review committee for new drugs for MDR-TB in the Republic of Korea. PHWR, 2017;10(50):1362-1368.
