# Supplementary material for: Tuberculosis control in the Republic of Korea
Source: Epidemiol Health. 2018 Aug 2;40:e2018036. doi: 10.4178/epih.e2018036 (PMC6335497; doi:10.4178/epih.e2018036)
Supplement: Supplementary file 10 [file epih-40-e2018036-supplementary9.pdf]

Supplementary Material 9

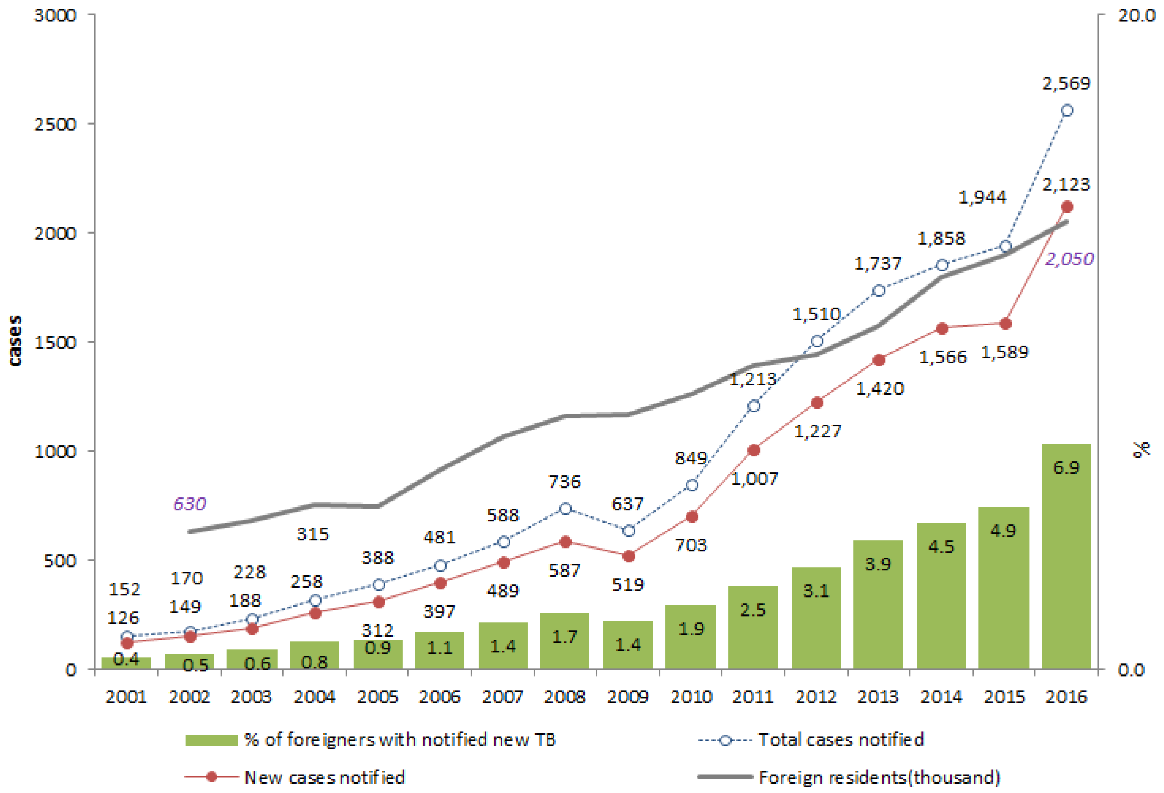

Original Source: 1) KCDC. 2016 Annual Report on the Notified Tuberculosis in Korea. Osong: Korea Centers for Diseases Control and Prevention; 2017.

2) Ministry of Justice. Korea immigration statistics. Kwacheon: Ministry of Justice; 2002; 2009; 2013; 2016.

Source: Cho KS. Tuberculosis Control in the Republic of Korea. Health and Social Welfare Review 2017;37(4):179-212.

Figure S5. Number of notified TB cases and proportion among foreigners by year.
