# Supplementary material for: Tuberculosis control in the Republic of Korea
Source: Epidemiol Health. 2018 Aug 2;40:e2018036. doi: 10.4178/epih.e2018036 (PMC6335497; doi:10.4178/epih.e2018036)
Supplement: Supplementary file 11 [file epih-40-e2018036-supplementary10.pdf]

Supplementary Material 10

Table S5. Age specific rates of positive LTBI by type of LTBI test

unit: %, yrs

Age group	Rate of positive LTBI by IGRA in 20171)*	Rate of positive LTBI by TST in 20162)**
10-19	n.a.	6.5
20-29	5.5	10.9
30-39	12.7	36.4
40-49	23.0	46.1
50-59	35.1	48.7
60-64	42.0	45.0
Total	20.3	33.2
Mean age of of individuals tested	41.0	40.7
Mean age of positive LTBI	47.9	n.a.

note: IGRA, Interferon-gamma releasing assay; TST, tuberculin skin test.

* Results from 543,538 facility workers including health care workers, nursery workers, and workers in children and other welfare facilities.

** Results from general population (n=2,051).

Source: 1) Cho KS, Park WS, Jeong HR, Kim MJ, Park SJ, Park AY, et al. Prevalence of latent tuberculosis infection at congregated settings in the Republic of Korea, 2017. KCDC PHWR 2018;11(12):348-354.

2) KCDC, KIT. 7th Korea National Health and Nutrition Examination Survey 1st year (2016) Tuberculin Survey Support and Quality Control. Osong: Korea Centers for Diseases Control and Prevention, Korean Institute of Tuberculosis; 2016.
