# Supplementary material for: Tuberculosis control in the Republic of Korea
Source: Epidemiol Health. 2018 Aug 2;40:e2018036. doi: 10.4178/epih.e2018036 (PMC6335497; doi:10.4178/epih.e2018036)
Supplement: Supplementary file 12 [file epih-40-e2018036-supplementary11.pdf]

Supplementary Material 11

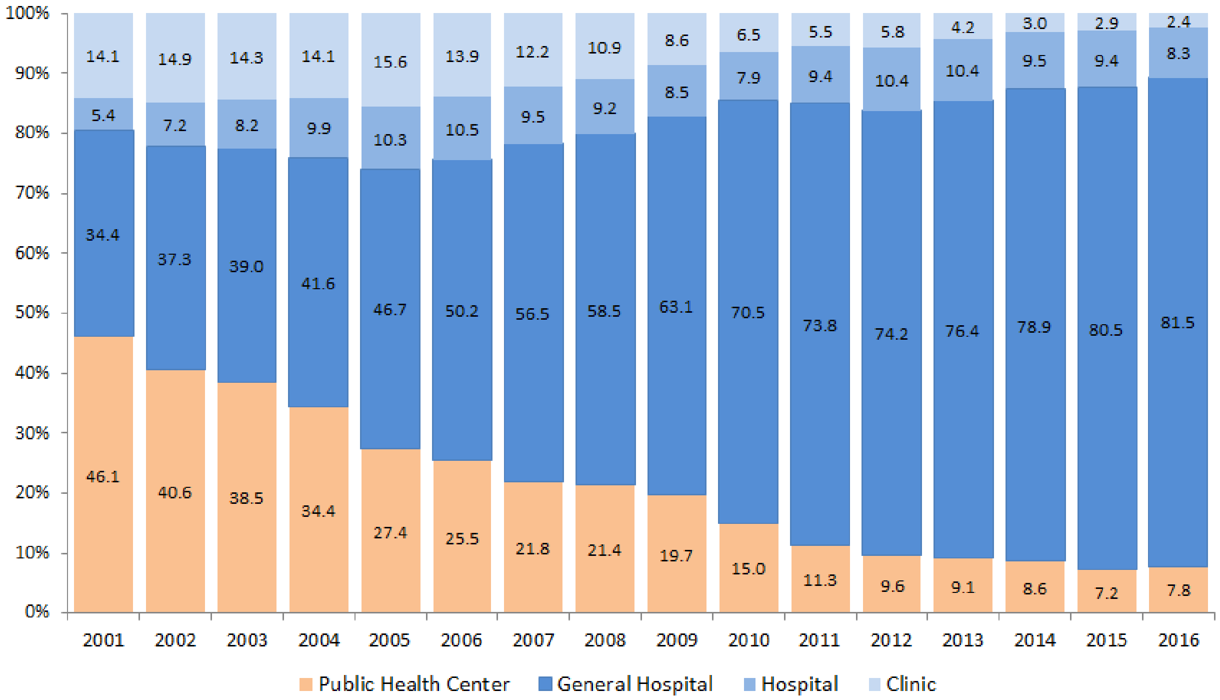

Source: KCDC. 2016 Annual Report on the Notified Tuberculosis in Korea. Osong: Korea Centers for Diseases Control and Prevention; 2017.

Figure S6. Proportion of new TB cases notified by type of heath care institution
